# Referential Dependencies Between Conflicting Attitudes

**DOI:** 10.1007/s10992-016-9397-7

**Published:** 2016-04-15

**Authors:** Emar Maier

**Affiliations:** University of Groningen, Oude Boteringestraat 52, 9712GL Groningen, The Netherlands

**Keywords:** Propositional attitudes, Hans Kamp, Ninan’s puzzle, DRT, Dynamic semantics

## Abstract

A number of puzzles about propositional attitudes in semantics and philosophy revolve around apparent referential dependencies between different attitudes within a single agent’s mental state. In a series of papers, Hans Kamp (2003… 2015) offers a general framework for describing such interconnected attitude complexes, building on DRT and dynamic semantics. I demonstrate that Kamp’s proposal cannot deal with referential dependencies between semantically conflicting attitudes, such as those in Ninan’s (21) puzzle about *de re* imagination. To solve the problem I propose to replace Kamp’s treatment of attitudes as context change potentials with a two-dimensional analysis.

## Three puzzles about dependent attitudes

Detective Mary investigates a mysterious death. She thinks the deceased was murdered and she hopes that the murderer is soon caught and arrested. A standard analysis of definite descriptions and of hope as a propositional attitude gives us two different ways of characterizing Mary’s hope that the murderer is arrested (Quine [24]):^1^

1.
  - *de dicto* : HOPE_m_∃x[∀y[murderer(y)⇔x=y]∧arrested(x)]
  - *de re* : ∃x[∀y[murderer(y)⇔x=y]∧HOPE_m_arrested(x)]

Neither of these logical forms captures what’s going on in the scenario. The *de re* construal in (1b) is out since Mary’s hope is not directed towards a specific individual she is acquainted with, but rather towards whoever committed the murder. The *de dicto* construal in (1a) is out because it entails that Mary hopes that there is a murderer, while intuitively the scenario should be compatible with Mary desiring a world without murder.^2^ What is going on, I submit, is that *relative to* her (*de dicto*) belief that someone murdered the victim, Mary hopes that he or she is arrested. In other words, Mary’s hope crucially depends on her beliefs.

Another illustration of the phenomenon of non-doxastic attitudes depending on beliefs, comes from the interaction between attitude ascriptions and presuppositions in discourse. Karttunen [17] observes that presuppositions triggered inside a hope ascription can be satisfied by a preceding belief ascription.

(2) Bill believed that Fred had been beating his wife and hoped that Fred would stop beating her.

This projection behavior is unexpected on the classical analysis of hopes and beliefs as propositional attitudes. Linguistic analyses of Karttunen’s puzzle again appeal to the idea of buletic attitudes being dependent on doxastic attitudes. Heim [10], for instance, defines wanting in terms of a preference ranking on doxastic alternatives to derive this peculiar projection behavior.^3^

A third puzzle concerns the standard analysis of *de re* attitudes in terms of acquaintance Lewis [18]:

(3) a believes *de re* of b that he is P iff there is an acquaintance relation R with R(a,b) and BEL_a_∃x[∀y[R(i,y)⇔y=x]∧P(x)].

In words, Mary believes *de re* of John that he’s an idiot iff Mary is in fact acquainted with John, say, as a TV celebrity she saw on a talkshow once, and she believes (*de dicto*) that the person she herself (the *de se* self-concept is represented as i in (3))^4^ is acquainted with, i.e., the celebrity she saw on TV, is an idiot.

Ninan [21] constructs a clear counterexample to a straightforward extension of this analysis of *de re* belief to *de re* imagination. He sketches the following scenario:

(4) Ralph sees Ortcutt sneaking around at the docks. Assume that this is the only way in which he is acquainted with Ortcutt, i.e., he has never been in any epistemic contact with this guy ever before. Based on his acquaintance with Ortcutt he can start to imagine different things about him. For instance, he can imagine what it would be like if he had never crossed paths with him.

According to the generalized Lewisian account we would capture this *de re* imagination as follows:

(5) ∃R[R(r,o)∧IMG_r_∃x[∀y[R(i,y)⇔y=x]∧never.cross.paths(i,x)]]

But that means that, given the acquaintance relation R between Ralph and Ortcutt, in all of Ralph’s imagination alternatives he himself (i.e., the *de se* center i) is R-acquainted with someone with whom he has never crossed paths. But that amounts to Ralph imagining a contradiction: being R-acquainted with someone while never having crossed paths with them. We can construct similar examples with other counterfactual attitudes – e.g., wishing or pretending that the thing you are looking at never existed.^5^

As with the previous two puzzles, a parasitic analysis of imagination as dependent on a doxastic background would solve the problem. Roughly, Ralph is then said to believe that he is R-acquainted with someone and imagine, relative to that belief, that he never met *that person*.

## Aims and Scope

Hans Kamp ([12–16]) has been developing a framework for formally representing mental states. Although not explicitly designed to solve these particular puzzles, it is ideally suited for dealing with parasitic attitudes generally. By way of a preview of the formal system that I will introduce, defend, and modify below, let me demonstrate what the internal mental states of the protagonists of these puzzles would look like in a (simplified fragment of) Kamp’s framework. In a nutshell, mental states are sets of attitudes, which in turn consist of a mode indicator (BEL for belief, DES for desire, etc.), and a Discourse Representation Structure (DRS) specifying the content of the attitude in question. I’ll use the standard two-dimensional, box representation of DRSs in which (i) an (optional) top compartment marks the introduction of some discourse referents, think of this as existential quantification over variables, and the bottom compartment contains descriptive conditions on these discourse referents. Concretely, the mental states of detective Mary, Karttunen’s Fred, and Ninan’s Ralph will be represented as follows:

(6)

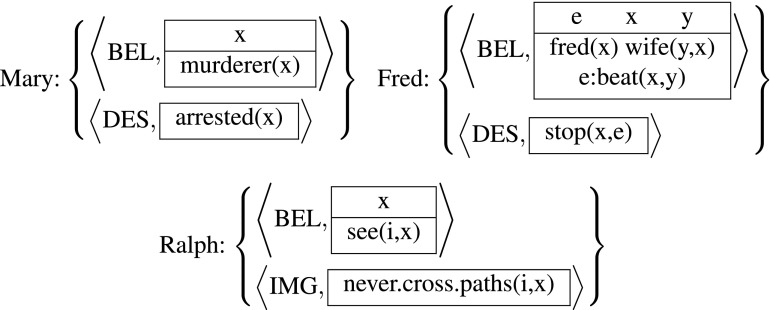

Crucially, in all three mental states a non-doxastic attitude is dependent on a doxastic attitude in the sense that the non-doxastic attitude contains conditions with a discourse referent that is introduced only in the doxastic attitude. This sharing of discourse referents across attitudes allows us to capture the parasitic nature of the puzzling attitudes, as diagnosed informally in Section 1. Thus the aim of this paper is to provide a precise and general syntax and semantics for mental state representations like the ones in (6).

### Attitudes vs. Reports

It is important to note that the so-called Attitude Description Sets (ADS) in (6) are *not* intended to directly represent the truth conditions of attitude reports, i.e., sentences like *Mary hopes that the murderer is arrested*, but to represent the (relevant parts of the) mental states of the protagonists of our puzzles. Formulating a linguistic semantics of reports and a theory of mental states are distinct endeavors, but they are not independent. On the basis of the theory of mental representation developed in this paper we could eventually develop a semantics of attitude reports, in DRT or some other framework. The first step in this extension from attitudes to reports would be a rule that says that a statement of the form ‘ *α* believes/hopes/imagines that *ϕ*’ is true iff *α* has a mental state that is captured by an ADS in which the believe, hope, or imagination component, respectively, contains the information contributed by *ϕ*.

Of course, this is just a rough first step towards a natural language report semantics. An empirically adequate, compositional implementation faces many challenges that I will not go into here.^6^ What does concern us here is what kind of structure to put in our mental state representations (i.e., the syntax of our ADSs), and what it means for an ADS to “capture” a mental state (i.e., the semantics).

### Referential Dependence vs. Intentional Identity

Distinguishing attitudes from reports also brings out an important difference between the parasitic attitudes under discussion and the seemingly related phenomenon of intentional identity, as exemplified, first and foremost, by Geach’s [7] Hob–Nob sentence.

(7) Hob thinks a witch has blighted Bobs mare and Nob wonders whether she killed Cob’s sow.

In (7), the pronoun *she* in the complement of the attitude verb *wonders* appears to be bound by *a witch* introduced in the complement of *thinks*, just like in (2) the presupposition triggered in the complement of *hoped* is apparently bound by the information contributed under the earlier *believed*. However, a closer look at the attitudes ascribed to the protagonists in (7) and (2) reveals that Nob’s wondering is not parasitic or referentially dependent on Hob’s beliefs in the way that Bill’s hope is parasitic on his belief. Despite the anaphoric dependency in the Hob–Nob report, the underlying attitudes of Hob and Nob are actually independent in the sense that (7) can be true even if Nob knows nothing about Hob and his thoughts, or about Bob and his mare. In the Karttunen puzzle, by contrast, the hope is dependent on the belief in the strong sense that Bill’s hope cannot stand on its own or even be formulated properly without recourse to the belief.

Non-reportative examples of intentional identity, as discussed in particular by Dekker [1, 2], exhibit a similar independence as the Hob–Nob reports.

(8) A: A man is sleeping over there on a park bench.

    B: It is not a man, it is a woman and she is not asleep, she is just sunbathing.

    Besides, it is not a park bench. [Dekker & van Rooy 1997:4]

Superficially, it seems as if the belief expressed by B is referentially dependent on that expressed by A, because the pronouns *it* and *she* in her response are bound by discourse referents introduced by indefinites in A’s utterance. Moreover, just as in Ninan’s puzzle, (4), these anaphorically linked attitudes are semantically incompatible. However, although the particular form in which B chose to express his attitude only works in the context of A’s utterance, the *de re* attitude itself is not strictly speaking dependent on anything in A’s mental state and could equally well be expressed independently of it, with demonstratives or other directly referential expressions instead of anaphoric pronouns. I conclude that intentional identity is a distinct phenomenon from the parasitic attitudes in the puzzles above.

In sum, this article is about how to model referential dependencies between different attitudes within a single agent’s complex mental state. I leave the semantics of attitude reports and intentional identity for another occasion.

The remainder of the paper is structured as follows. In Section 3 I first guide the reader through a basic fragment of Kamp’s theory of mental representation. In Section 4 I argue that Kamp’s semantics fails to account for referential dependencies between conflicting attitudes, like in Ninan’s puzzle and non-*de re* variations thereof. In Section 5 I propose a modification of Kamp’s mental state semantics that addresses the generalized Ninan puzzle with a two-dimensional semantics for non-doxastic attitudes.

## Representing Mental States

As illustrated in (6), the basic idea is to represent mental states as Attitude Description Sets (ADS), sets of formulas in the DRS language paired with mode indicators like BEL, DES, or IMG, specifying what the agent believes, desires and imagines, respectively.

In this section I reconstruct the ADT framework presented most comprehensively in Section 5 of the (book-length) DRT survey in the *Handbook of Philosophical Logic* (Kamp et al. [16]). To focus the discussion I introduce some minor simplifications. In particular, I restrict attention to the three modes already encountered above. Thus, I ignore the special mode indicator ANCH, that Kamp uses in his articles to mark internal anchors. For Kamp, internal anchors are descriptive representations of external *res* that the agent is acquainted with. In particular, Kamp’s representation of the *de re* imagination in Ninan’s puzzle would involve an ANCH[x]-label rather than a BEL-label on the box with the acquaintance information. Internal anchors, and the corresponding external anchors that they presuppose, are indispensable in Kamp’s semantic analysis of indefinites [15], proper names [14], and *de re* attitudes and ascriptions [13]. However, as far as capturing the ‘narrow’ psychological interpretation of ADSs is concerned, anchors in Kamp’s semantics really just amount to beliefs. Since the puzzles above are about properly describing the internal mental states of the protagonists involved, I will simply replace anchors with beliefs in my ADSs, as already illustrated in the final ADS in (6).

Below I will first introduce some basic DRT notions, including its dynamic semantics in terms of information states and context change potentials. With these notions in place I then reconstruct Kamp’s ADS semantics. For readability, formal details are moved to an Appendix. Readers familiar with DRT and dynamic semantics may want to skip Subsection 3.1.

### Discourse Representation Theory (DRT)

#### Introducing DRT

Kamp [11] introduced Discourse Representation Theory (DRT) as a semantic theory of anaphora resolution in discourse. Its main selling points at the time were its solution to two problems that plagued Montague Grammar, the dominant semantic framework at the time, viz. cross-sentential anaphora (9a) and donkey sentences (9b).

(9) a. A friend of mine owns a donkey. He beats it.

    b. If a farmer owns a donkey, he beats it.

The problem with both of these is that intuitively *he* and *it* are variables bound by the existential quantifiers in *a farmer*/*a friend of mine* and *a donkey*, respectively, but a static, compositional semantics like Montague’s cannot derive plausible readings where this is so.^7^ For (9a), binding is out because to interpret the discourse we must start with the first sentence, and when that’s done we have a closed formula:

(10) ∃x∃y[friend(x)∧donkey(y)∧own(x,y)]

Note that the scope of the quantifiers in (10) is delimited by a closing bracket on the right, so subsequent occurrences of x and y would be free.

For (9b) the problem is that indefinite noun phrases like *a farmer* always introduce existential quantifiers into the compositional derivation, and even if we allow those to take wide scope over the conditional (which Montague would in fact allow), this never gives the right truth conditions.

Kamp’s solution involves a radical move, from a compositional, truth-conditional sentence semantics to a dynamic discourse interpretation algorithm. The sentences in a discourse are interpreted as successive updates on a context, represented as a DRS. In other words, a sentence is analyzed as providing instructions on how to turn an input DRS into an updated, more informative output DRS, capturing the Stalnakerian [26] idea that interpreting a sentence induces a contextual information growth. In the update process, there is a fundamental split between indefinites, which introduce new discourse referents into the context, and definites, including pronouns, which pick up previously established, old discourse referents. Note finally that it is the input and output DRSs, not the sentences, that are semantically interpreted in a traditional model and that express the truth conditions of the discourse up to a certain point.

Let me illustrate the above with the discourse in (9a). We start with an empty DRS when the first sentence comes in. The two indefinites introduce fresh discourse referents x and y into the DRS, resulting in the following output:

(11)

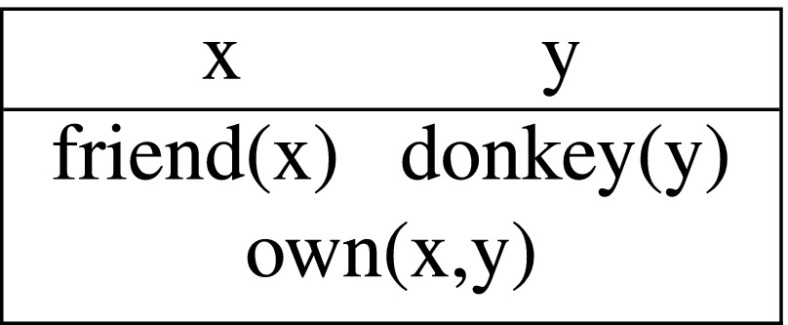

This output DRS represents the information that there is farmer and a donkey and the former owns the latter, i.e., (11) is equivalent to (10) (as we can verify on the basis of the precise syntax and semantics defined below). The crucial difference with the static system comes out with the second sentence. The pronouns are interpreted as instructions to retrieve appropriate antecedents from the input context. Since *he* requires a human antecedent, and *it* a non-human, the only option is to bind the first to x and the second to y:

(12)

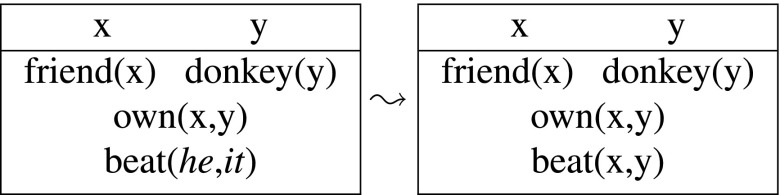

This final output DRS says that there is a friend of mine and a donkey and that friend owns and beats that donkey, which corresponds to the intuitive truth conditions of the discourse in (9a).

Kamp’s solution to the donkey anaphora puzzle in (9b) relies on a dynamic interpretation of conditionals. Crucially, the indefinites and pronouns are treated exactly as in the previous example: *a farmer* and *a donkey* introduce new discourse referents, while the pronouns are looking for previously established antecedents. Given the DRT syntax and semantics of conditionals, formally defined in Sections 3.1.2–3.1.3 below, discourse referents introduced in the conditional antecedens can bind variables in the consequens, so (13) is a well-formed, interpretable output:

(13)

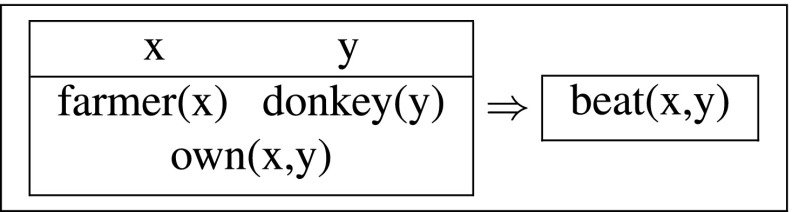

With the semantics for ⇒ (defined in (15c) below), this output DRS expresses the right truth conditions, viz. that for every farmer *f* and donkey *d*, if *f* owns *d*, *f* also beats *d*.

#### The Syntax of DRT

A DRS *K* is a pair, 〈*U*(*K*),*C* *o* *n*(*K*)〉, consisting of a “universe” of discourse referents (x,y, …), and a set of conditions. Conditions can be atomic (e.g. walk(x)) or complex (e.g. ¬*K* ^*′*^, with *K* ^*′*^ a DRS). Graphically, I’ll continue to represent a DRS as a box with two compartments; the top compartment represents the universe, the bottom the conditions (but I suppress the top compartment if it’s empty).

We (recursively) define *F* *V*(*K*), the free variables (or free discourse referents) of *K*, as the set of discourse referents that occur free in the conditions but not in the universe of *K*. DRSs without free variables we call proper. For details, see the Appendix.

#### A Static Semantics for DRT

DRT provides a dynamic theory of interpretation, i.e., utterances are analyzed as inducing information growth, modeled by means of DRS updates. Nonetheless, DRSs are typically given a static semantics in the form of a truth definition. Here is an intensional version. Intensional models have the form $\left \langle D,W,\mathbb {I} \right \rangle $, where *D* is a domain of individuals, *W* a set of possible worlds, and $\mathbb {I}$ an interpretation function that assigns sets of *n*-tuples of individuals to *n*-ary predicates, relative to a possible world parameter.

The central notion of DRT semantics is that of a verifying embedding. A verifying embedding of a proper DRS is an assignment function that maps all the discourse referents in the universe of the DRS to entities in the model’s domain in such a way that all conditions in that DRS come out true:

(14) *f*⊧_*w*_ *K* iff for all *ψ*∈ *C* *o* *n*(*K*):*f* ⊧_*w*_ *ψ*.

Condition verification is defined by cases. For example:

(15) a. $f\models _{w} P(x_{1},{\ldots } x_{n})\, \text {iff}\, \left \langle f(x_{1}),\ldots ,f(x_{n}) \right \rangle \in \mathbb {I}_{w}(P)$

     b. *f*⊧_*w*_¬*K* ^*′*^ iff there is no *g*⊇*f* with *D* *o* *m*(*g*) = *D* *o* *m*(*f*)∪*U*(*K* ^*′*^) and *g*⊧_*w*_ *K* ^*′*^.

     c. *f*⊧_*w*_ *K* ^*′*^⇒*K* ^*′′*^ iff for all *g*⊇*f* with *D* *o* *m*(*g*) = *D* *o* *m*(*f*)∪*U*(*K* ^*′*^) and *g*⊧_*w*_ *K* ^*′*^,there is an *h*⊇*g* with *D* *o* *m*(*h*) = *D* *o* *m*(*g*)∪*U*(*K* ^*′′*^) and *h*⊧_*w*_ *K* ^*′′*^.

A proper DRS, i.e., a DRS without free variables, is true in *w* if there exists a verifying embedding of its universe into *D*. This leads to the following definition of the proposition expressed by a proper DRS *K*.

(16) [[*K*]]={*w*∈*W* there is an *f* with *D* *o* *m*(*f*) = *U*(*K*) and *f*⊧_*w*_ *K*}

This gives us a static interpretation of proper DRSs with which I will emulate a classical Hintikka/Kripke analysis of propositional attitudes as intensional operators in Section 3.2.2. As I will show there, such a classical semantics can’t account for the referential dependencies in the ADSs exemplified in (6) above. To solve this Kamp switches to a dynamic DRS semantics, mapping DRSs to information states and context change potentials. Before turning to attitudes I introduce here already the required notions from dynamic semantics.

#### A Dynamic Semantics for DRT

The starting point of dynamic semantics is that the context is modeled as an information state and a sentence’s meaning is a function that transforms such a context into a new, more informative one. Formally, information states are just sets of *possibilities*, i.e. world–assignment pairs. Proper DRSs express information states.

(17) [[*K*]]^is^={〈*w*,*f*〉 *D* *o* *m*(*f*) = *U*(*K*) and *f*⊧_*w*_ *K*}

[[*K*]]^is^ captures the informational content of a proper DRS in a strictly more fine-grained way than [[*K*]] does, in the sense that from an information state we can reconstruct the proposition (by deleting all the assignment functions) but not the other way around. In other words, the information state expressed by *K* gives you for every world in which *K* holds the embeddings that verify it, while a classical proposition expressed by *K* only tells you for each world whether there exists at least one verifying embedding.

To illustrate the key notion of an information state, consider the first example DRS from Subsection 3.1.1, repeated below:

(18)

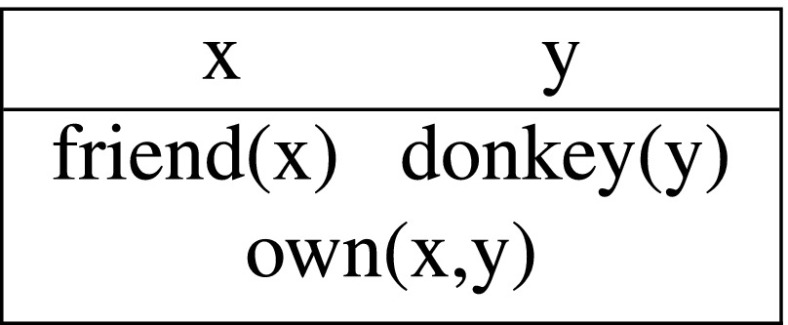

The proposition expressed by (18), [[(18)]], is the set of worlds in which there exists an embedding verifying the three conditions in *w*, i.e. an embedding *f* with *f*(x) is a friend of mine in *w*, *f*(y) is a donkey in *w*, and *f*(x) owns *f*(x) in *w*. In other words, the set of worlds in which there exist at least one friend who owns at least one donkey. The information state expressed by (18), (18) ^is^, is the set of all possibilities 〈*w*,*f*〉 for which *D* *o* *m*(*f*)={x,y}, and *f* verifies the three conditions in *w*.

The truly dynamic content of a DRS should model the way it affects its context, modeled as an information state, i.e., its context change potential. Below I’ll formalize context change potentials as partial functions from input to output information states. However, before going into the technical details, I should point out that my eventual proposal does not depend on the notion of a context change potential. The only reason I discuss it here is in order to be able to reconstruct Kamp et al.’s [16] proposal, which, I will argue, is ultimately unsatisfactory. The reader who is not interested in my reconstruction of Kamp’s proposal (Subsection 3.2.3) and why it fails (Section 4) could safely skip the remainder of this section as well. For a proper DRS *K* the idea is that [[*K*]]^ccp^ extends its input information state with information from *K*, by extending the verifying embeddings to cover *U*(*K*) and throwing out possibilities not compatible with *C* *o* *n*(*K*). More precisely, for a proper DRS *K* and an information state *s*:

(19) [[*K*]]^ccp^(*s*)={〈*w*,*f*〉 ∃*g*[〈*w*,*g*〉∈*s*∧*f*⊇*g*∧*D* *o* *m*(*f*) = *D* *o* *m*(*g*)∪*U*(*K*) ∧*f*⊧_*w*_ *K*]}

An improper DRS *K* updates a context in the same way, but only if that context introduces the discourse referents that are free in *K*. To make this precise note first that in an information state expressed by a proper DRS all embeddings have the same domain. We call this domain the base of the information state. In fact, throughout the paper we’ll only consider information states *s* that have a well-defined base *B* *a* *s* *e*(*s*). We can now say that [[*K*]]^ccp^ is only defined for an information state *s* if *F* *V*(*K*)⊆*B* *a* *s* *e*(*s*); if defined, its value is given by (19).

### Attitude Description Theory (ADT)

#### The Syntax of ADT

An Attitude Description Set (ADS) is a set of pairs of the form 〈*M*,*K*〉, where *M* is a mode label (e.g., BEL, DES, IMG) and *K* a DRS. We’ve already seen that some of the attitude DRSs contain free variables. In a proper ADS all free variables in these open DRSs are introduced in some other DRS. If a free variable in *K*_1_ is introduced in the universe of *K*_2_ we say that *K*_1_ is referentially dependent on *K*_2_, notation: *K*_2_≺*K*_1_. For instance, in the simple examples of ADSs considered in (6) above we’ve seen desire DRSs and an imagination DRS referentially dependent on belief DRSs.

An ADS $\mathcal {K}$ is well-founded if all chains of such referential dependence are non-circular, i.e. if the transitive closure of ≺ on $\mathcal {K}$ is well-founded. In the following we exclude ADSs as in (20) which are either improper (20a), or not well-founded (20b).

(20)

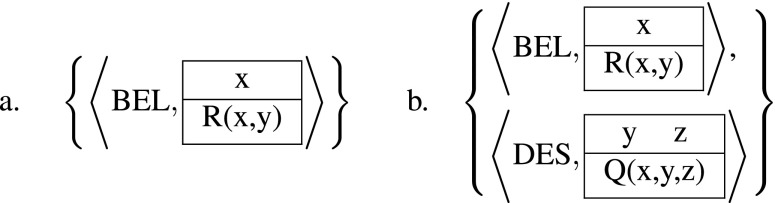

A distinctive feature of Kamp’s analysis is that mental state descriptions may contain multiple distinct attitudes even of the same mode, as illustrated in (21):

(21) {〈 BEL,*K*_1_〉,〈 BEL,*K*_2_〉,〈 DES,*K*_3_〉}, with *K*_*i*_ DRSs.

Kamp’s primary motivation for representing beliefs as discrete entities rather than specifying just a single belief state seems to be to avoid some instances of the problem of logical omniscience. Specifically, the type exemplified by Lewis’s [19] inconsistent beliefs that Nassau Street runs roughly east–west, that the railroad runs north–south, and that the two are roughly parallel. To avoid ascribing to himself a single inconsistent belief state, Lewis proposes that a belief state may be “fragmented”, containing various distinct but overlapping belief fragments. We can think of the distinct BEL -labeled DRS in an ADS as representing such belief fragments. Apart from combating logical omniscience, the expressive power afforded by the fragmented attitudes model may be particularly valuable when developing potential refinements where we, for instance, assign different weights/probabilities to different beliefs and/or model fine-grained belief revision mechanisms.

Below I sketch Kamp’s semantics for the ADS language, and show what’s wrong with it, but to motivate the substantial machinery involved I’ll start with developing a classical propositional approach and see why that doesn’t work in the first place.^8^

#### A Static Semantics for ADT

Giving a semantics for ADT means defining when a given ADS accurately represents an agent’s mental state as given by the model. The first question is therefore, what kind of mental state entities do we add to our intensional models? And the second, how do we ‘match’ those with an ADS?

If we just focus on belief first, the classical (Kripke/Hintikka-style) answer to the first question would be to specify for each agent (at a time and world) a set of doxastic alternatives. The intuitive motivation is that a world *w* is a doxastic alternative for an agent if, were we to place that agent in *w* and allow her to investigate indefinitely, she would find no evidence that *w* was not her home world (Haas-Spohn [8]). The answer to the second question, i.e., the matching condition, is the requirement that x’s belief state is compatible with a sentence *ϕ* iff the proposition expressed by *ϕ* is true in all doxastic alternatives.

To extend this familiar picture to mental states consisting of multiple, distinct attitudes, we first add a set of a buletic alternatives, and a set of imagination alternatives. A mental state is now modeled as a triple of propositions.^9^ But in our fragmented model we have not one set of doxastic alternatives, but multiple potentially overlapping ones – and similarly for the other modes of attitude. Matching the syntax of ADSs we thus end up modelling an agent’s mental state as what I will call, mimicking the terminology of Kamp et al. [16], a Proposition-Based Attitudinal State description (PBAS), a set of propositions paired with mode indicators.

(22) {〈 BEL,*p*_1_〉,〈 BEL,*p*_2_〉,〈 DES,*p*_3_〉}, with *p*_*i*_⊆*W*.

Then the question of matching: When does a syntactic description in the form of an ADS like (21) count as an accurate representation of a semantic PBAS like (22)? The first observation is that if John’s mental state includes, say, a desire to buy a red Mercedes we want to say that an ADS representation of John with a desire to buy a car is correct. To capture this type of partial matching we say, following the Hintikka/Kripke analysis, that a syntactic ADS matches a semantic PBAS if the attitudes in the PBAS *entail* the contents of the corresponding attitudes represented in the ADS. The relevant notion of entailment here is the standard entailment between propositions: *p* entails *q* iff *p*⊆*q*. In sum, we model the interpretation of an ADS relative to a PBAS with the following matching condition:

(23) $\text {An ADS}\, \mathcal {K} \,\text {captures the PBAS}\, \mathcal {A}\, \text {iff for every}\, \left \langle M,K \right \rangle \in \mathcal {K}\, \text {there is a}$ $\langle {M,p}\rangle \in \mathcal {A}\, \text {such that}\, p\subseteq {K}]{\kern -2.3pt}].$

For some very simple examples where all attitude DRSs in an ADS are proper this will give adequate results. However, the whole point of the ADS structure is to allow referential dependencies between attitudes, which involves improper attitude DRSs (inside an overall proper ADS). Consider detective Mary’s mental state: she believes there is a murderer x, and desires that x get caught.

(24)

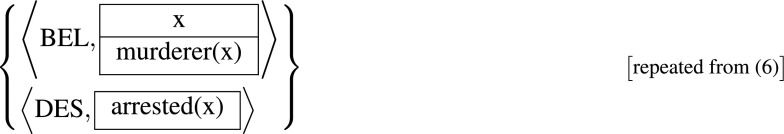

In other words, Mary’s desire DRS has a free variable x and hence does not express a classical proposition on its own.

Conclusion: the classical proposition-based approach fails because attitudes that are referentially dependent on other attitudes do not express propositions. Kamp’s actual proposal relies on a dynamic rather than static semantics, precisely because we can then assign appropriate semantic values to improper DRSs.

#### A Dynamic Semantics for ADT

Kamp’s alternative answer to the first question is that the model should associate with each agent an Information State Based Attitudinal State description (ISBAS), a set-theoretic construct in which an agent’s attitudes are represented as dynamic semantic values, i.e. context change potentials.

The move from propositions to context change potentials can be broken down into two steps. First, we move from propositions (sets of possible worlds) to information states (sets of world–assignment pairs). Both are ways of formalizing information, but, as noted above, the latter is strictly more finegrained. The extra structure in information states is used in dynamic semantics to account for anaphoric dependencies in discourse. Here, we exploit it to account for referential dependencies between attitudes. In a PBAS the set of worlds *w* in which there exist at least one farmer and one donkey such that the former beats the latter constitute the propositional belief that there is a farmer who beats a donkey. Here, by contrast, the set of possibilities of the form 〈*w*,*f*〉 with *f*(x) is a farmer in *w*, *f*(y) a donkey, and *f*(x) beats *f*(y) in *w* models the more finely individuated belief that a farmer mentally represented by the discourse referent x beats a donkey, represented as y. In other words, modeled as information states, beliefs and other attitudes carry information organized around specific (mental) discourse referents.^10^

Switching from propositions to information states however does not solve the problem identified above: a referentially dependent attitude DRS expresses neither a proposition nor an information state, so we still can’t directly match the DRSs in an ADS with corresponding information states in an ISBAS. To fix this, the second step is to switch from information states to context change potentials (CCP), which is a semantic notion designed to capture the dynamic content of any DRS, with or without free variables. This solves the matching problem for referentially dependent attitudes. Concretely, the agent’s complete mental state is now represented as a set of labeled CCPs, and an ADS captures an ISBAS if the labeled CCPs in the ISBAS entail the CCPs expressed by the corresponding attitude DRSs in the ADS.

A complication is that the relevant notion of entailment in dynamic semantics is defined at the level of information states, not context change potentials, viz. through the relation ▹:

(25) *s*▹*s* ^*′*^ iff s contains at least as much information as s ^*′*^ (where ‘more information’ would mean a larger base and/or fewer possibilities, cf. Appendix for definition)

Somehow we need to extract information states from the CCPs in the ISBAS, and from the (proper and improper) attitude DRSs in an ADS. The idea is the same in both cases, but let’s start with the ADS.

A proper attitude DRS already expresses an information state but an improper one does not. It expresses merely a partial context change potential, which needs a suitable input information state to provide a information state that captures its content. In well-founded and proper ADSs any improper attitude DRS referentially depends on other attitude DRSs, and these may again depend on others, but eventually all free variables will be grounded in proper DRSs. To illustrate the general framework consider an abstract example ADS with an imagination depending on a belief and a desire, which depends on another belief:

(26) Example ADS: {〈 BEL,*K*_1_〉,〈 BEL,*K*_2_〉,〈 DES,*K*_3_〉,〈 IMG,*K*_4_〉}, with *K*_1_≺ *K*_4_ and *K*_2_≺*K*_3_≺*K*_4_.

The DRSs at the bottom of the ≺-chains, *K*_1_ and *K*_2_, are independent and hence proper DRSs, which directly express information states. *K*_3_ is not independent and hence expresses only a CCP. We turn that CCP into a suitable information state by applying it to the information state expressed by the DRS it depends on within $\mathcal {K}$, i.e. using it as an update on the belief state expressed by *K*_2_:

(27) ${[{\kern -2.3pt}[ K_{3}]{\kern -2.3pt}]}^{\textsf {is}}_{\mathcal {K}} = {[{\kern -2.3pt}[{K_{3}}]{\kern -2.3pt}]}^{\textsf {ccp}}({[{\kern -2.3pt}[{K_{2}}]{\kern -2.3pt}]}^{\textsf {is}}).$

Note that the partial context change potential expressed by *K*_3_ is indeed defined for this input argument because, by the assumed ≺-structure (and properness/well-foundedness), all free variables in *K*_3_ are introduced in *U*(*K*_2_).

Attitude DRSs may also depend on multiple other attitude DRSs. *K*_4_ depends on both *K*_1_ and *K*_3_ so its context change potential will not be defined on the information states expressed by either alone. Kamp proposes to first merge the information states of the underlying DRSs (cf. Appendix):

(28) $[{\kern -2.3pt}[{K_{4}]{\kern -2.3pt}]}^{\textsf {is}}_{\mathcal {K}} = {[{\kern -2.3pt}[ K_{4}]{\kern -2.3pt}]}^{\textsf {ccp}}({[{\kern -2.3pt}[ K_{1}]{\kern -2.3pt}]}^{\textsf {is}}_{\mathcal {K}}\uplus {[{\kern -2.3pt}[ K_{3}]{\kern -2.3pt}]}^{\textsf {is}}_{\mathcal {K}})$

In this way, Kamp manages to associate information states even with improper attitude DRSs parasitic on one or more other attitude DRS within the same ADS.

We apply the same tricks to associate information states with the CCPs that make up our ISBAS. This requires first of all a semantic analogue of referential dependence, ≺^∗^, as a relation between context change potentials (cf. ). With the help of ≺^∗^ we recursively associate information states with the CCPs in an ISBAS. A total CCP *j* determines a corresponding information state by applying it to the empty information state Λ: $I\!S(j, \mathcal {A})=j(\Lambda )$. A non-total CCP referentially depends on other information states lower in the ≺^∗^-chain. Assuming that these are already associated with information states, we define $I\!S(j,\mathcal {A})$ as the application of *j* to the merge of these underlying information states. We require of an ISBAS that this recursive procedure for assigning information states is well-defined.

Now that we can associate information states with the attitude DRSs in a given ADS and with the CCPs in a given ISBAS, we are almost ready to state the matching condition. One last complication in moving from proposition matching to information state matching comes from the introduction of discourse referents on the model-theoretic side. The problem is that the discourse referents in an ADS are chosen completely independently from the discourse referents in the agent’s head, as modeled by the ISBAS. We solve this by introducing a variable renaming function (and demanding that ADSs employ discourse referents from a set that is entirely disjoint from those that occur in ISBAS).

Finally then, we can state Kamp’s matching condition:

(29) $\text {An ADS}\,\, \mathcal {K}\, \,\text {captures an ISBAS}\,\, \mathcal {A}\,\, \text {iff there is a variable renaming function}$ $r\,\, \text {on}\,\, \mathcal {K}\,\, \text {such that for every} \,\, \left \langle M,K \right \rangle \in \mathcal {K}\,\, \text {there is a}\,\, \left \langle M,j \right \rangle \in \mathcal {A}\,\, \text {such that}$ $I\!S(j,\mathcal {A})\rhd {[{\kern -2.3pt}[{r(K)}]{\kern -2.3pt}]}^{\textsf {is}}_{r(\mathcal {K})}$

In concrete applications below I’ll abstract away from the variable renaming issue.

## A Problem: Conflicts with Dependencies

Kamp’s attitude semantics allows us to model referential dependencies between different attitudes in an agent’s mental state. Kamp is surely not the first to recognize that attitudes may be dependent on other attitudes within a single mental state, but he seems to have been the first to implement this in a completely general way. To see the benefits of this generality, compare it to, say, Heim’s [10] semantics of belief-dependent wanting. The crucial ingredient is the idea that desire can be analyzed in terms of a preference ranking of doxastic alternatives, which indeed allows Heim to capture the dependence of desire on belief. But this solution doesn’t extend to, say, Ninan’s case of belief-dependent imagination, since imagination cannot be explicated in terms of a preference ranking. Kamp’s system, by contrast, allows us to describe arbitrarily complex chains of dependencies between attitudes, including attitudes depending on multiple other attitudes simultaneously.

Kamp’s analysis builds on insights from dynamic semantics, in particular it uses context change potentials and information states rather than propositions to model the basic semantic content of the different attitudes. The trickiest part was assigning information states to these context change potentials, viz., by recursively applying the dependent context change potentials to the information states on which they depend. As I argue below, it is this updating that causes trouble, which we can best bring out by analyzing cases where the content of a dependent attitude conflicts with the content of an attitude it depends on.

Consider a counterfactual attitude, more specifically one whose content contradicts the content of an attitude it depends on. Ninan’s puzzle provides an example, but in order not to get stuck on orthogonal issues involving *de re* and *de se* attitudes, let’s modify the detective example. Say Mary imagines what it would be like if whoever murdered the victim had chosen a different lifestyle and never murdered anyone.

(30)

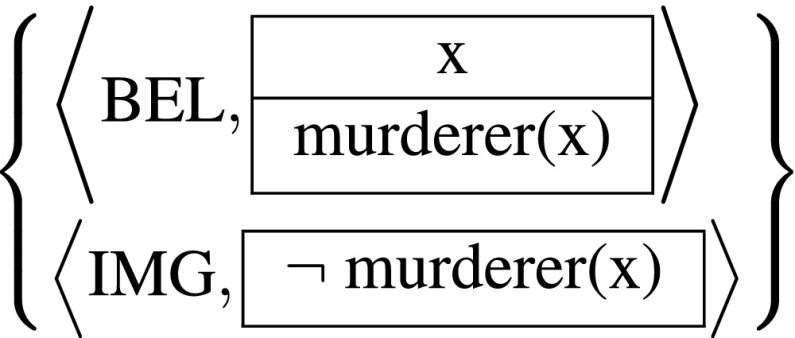

According to the Kampian semantics, this syntactic object $\mathcal {K}$ is a correct description of someone’s mental state at a certain time and world in an intensional model if the model’s ISBAS $\mathcal {A}$ at that time and world contains appropriately labeled CCPs that entail the corresponding dynamic semantic contents of the two DRSs in (30), which we will call *K*_1_ and *K*_2_. That is, if we can find in $\mathcal {A}$ a BEL-labeled CCP, *j*_1_, and a IMG-labeled CCP, *j*_2_, such that:

(31) a. $I\!S(j_{1}, \mathcal {A})\rhd [{\kern -2.3pt}[{K_{1}]{\kern -2.3pt}]}^{\textsf {is}}_{\mathcal {K}}$

     b. $I\!S(j_{2}, \mathcal {A})\rhd [{\kern -2.3pt}[{K_{2}]{\kern -2.3pt}]}^{\textsf {is}}_{\mathcal {K}}.$

To unpack (31a) (i.e., the matching condition for the belief component in (31)), we must determine the information states associated with *K*_1_ (in $\mathcal {K}$) and *j*_1_ (in $\mathcal {A}$):

(32) a. ${[{\kern -2.3pt}[ K_{1}]{\kern -2.3pt}]}^{\textsf {is}}_{\mathcal {K}} = \left \{\left \langle w,f \right \rangle \ \, \vline \,Dom(f)=\left \{\mathrm {x}\right \}\, \text {and}\, f(\mathrm {x})\, \text {is a murderer in}\, w \right \}$

     b. $I\!S(j_{1}, \mathcal {A}) = j_{1}(\Lambda )$

The matching requirement for the belief says that the ADS in (30) captures the agent’s beliefs iff the state in (32b) contains at least as much information as that in (32a). In words, this means that the doxastic CCP *j*_1_ in $\mathcal {A}$ must introduce (possibly among other things) a discourse referent x representing a murderer.

Now we similarly unpack (31b). The CCP expressed by *K*_2_ is a partial function on information states, which models the addition of the information in *K*_2_ to an input state that already provides the discourse referent x:

(33) For all *s* with x∈ *B* *a* *s* *e* *(* *s* *)*:[[*K*_2_]]^ccp^(*s*)={〈*w*,*f*〉∈*s* *f*⊧_*w*_ *K*_2_}={〈*w*,*f*〉∈*s* *f*(x) is not a murderer in *w*}

We compute the information state associated with *K*_2_ by applying this CCP to the belief state expressed by *K*_1_. The result is an inconsistent information state:

(34) ${[{\kern -2.3pt}[{K_{2}}]{\kern -2.3pt}]}^{\textsf {is}}_{\mathcal {K}} = {[{\kern -2.3pt}[{K_{2}}]{\kern -2.3pt}]}^{\textsf {ccp}}({[{\kern -2.3pt}[{K_{1}}]{\kern -2.3pt}]}^{\textsf {is}}_{\mathcal {K}}) = \{\ \left \langle w,f \right \rangle \ \vline \ \! Dom(f)= \left \{\mathrm {x}\right \}$ and *f*(x) is a murderer in *w* and *f*(x) is not a murderer in *w* } = *∅*

This seems like an undesirable result. The informational content of the imagination in the context of the belief it depends on should not be a trivial, contradictory information state. What we have derived here is that the ADS in (30), apparently describing a perfectly reasonable imagination, is semantically equivalent to one that describes an internally inconsistent imagination, e.g. that x is not a murderer but murdered someone, or simply that 0 =1.

Just to be sure, let’s finish spelling out the matching condition for imagination to convince ourselves that this will not do. First, we get the information state associated with *j*_2_ by applying it to the belief state associated with *j*_1_.

(35) $I\!S(j_{2}, \mathcal {A}) = j_{2}(I\!S(j_{1}, \mathcal {A}), \mathcal {A})$

The matching condition for imagination says that *K*_2_ captures the agent’s imagination if (35) entails (34), i.e., if the imagination CCP *j*_2_ is such that applying it will transform the belief state given by the ISBAS (that there is, possibly among other things, some x who is a murderer) into the inconsistent information state in (34) (that there is an x who is a murderer and not a murderer). That is, *j*_2_ matches *K*_2_ if it explodes the previously computed doxastic information state.

Now note that it is not unexpected as such that updating the belief in the ADS above with the imagination leads to inconsistency. The problem is rather that this inconsistency doesn’t tell us much about what the agent’s imagination, *j*_2_, is like. An internally inconsistent *j* that has nothing to do with *K*_2_ (e.g., the CCP that updates an input state *s* with the information that x ≠x) would also satisfy the matching condition for *K*_2_. To put it differently, the current system fails to distinguish the perfectly rational mental state of imagining something counter to what you believe, from a mental state with a truly incoherent imagination, such as that x ≠x.

## A Solution: Two-Dimensional Attitudes

### Ninan on *de re* imagination

Ninan [21] offers two distinct solutions to his puzzle. The first involves relativizing an agent’s imagination alternatives to a doxastic alternative, i.e., the model provides us with belief-relative-imagination as a new primitive. The second involves multi-centered worlds (further developed in Ninan [22]). In this Section I will show how we can generalize Ninan’s first proposal and adapt it to our dynamic setting to overcome the problem of conflicting attitudes in ADT.

Ninan’s idea is to add a second dimension to the notion of imagination. The model has to provide imagination alternatives relative to beliefs. One way to define the new belief-relative notion of imagination is as a function *I* *m* *g* from possible worlds to sets of possible worlds, i.e., what Ninan calls a “two-dimensional intension”.^11^

(36) *w* ^*′*^∈*I* *m* *g*(*w*) iff if the agent’s doxastic state were {*w*} (i.e. the agent believes she inhabits *w* and is maximally opinionated on the matter),then *w* ^*′*^ would be compatible with what the agent imagines.

To solve the *de re* imagination puzzle, Ninan then defines:

(37) *a* imagines *de re* of *b* that he is *P* iff there is an acquaintance relation *R* with: (i) *R*(*a,b*) and (ii) for all doxastic alternatives *w* there is a unique *b* ^*′*^ that *a* is *R*-acquainted with in *w*, and (iii) in all *w* ^*′*^∈*I* *m* *g*(*w*), *b* ^*′*^ is *P*.

In the scenario sketched in Section 1, *R* must be something like the relation *x sees y at the docks*, so what (37) says is that for each doxastic alternative *w* there is a unique man *b* ^*′*^ that *a* sees at the docks, and all (*w*-relative) imagination alternatives *w* ^*′*^ are such that *a* never met *b* ^*′*^. In sum, if we accept the new primitive belief-relative notion of imagination, we can straightforwardly solve Ninan’s puzzle.

Below I will incorporate a generalization of Ninan’s proposal for arbitrary attitudes into the dynamic ADT framework in order to solve the problem of conflicting dependent attitudes, as brought out for Kamp’s original ADT in Section 4 above.

### Two-Dimensional Attitudes in ADT

In my definitive proposal we model mental states model-theoretically as Ninan-Based Attitudinal State descriptions (NBAS). An NBAS contains two-dimensional attitude representations modeled after *I* *m* *g* from (36). More specifically, a two-dimensional attitude *Q* is a function mapping possibilities to information states such that *Q*(〈*w*,*f*〉) is the informational content of the attitude that the agent would have were her background beliefs captured by the singleton information state {〈*w*,*f*〉}. Note that, in dynamic semantics, a singleton information state is simply a maximally specific body of information about the relevant discourse referents. In other words, being in a singleton belief state means you are maximally opinionated about the world you inhabit and about the discourse referents the belief state is about.

We’ll assume that for any two-dimensional attitude *Q* all possibilities in *Q*(〈*w*,*f*〉) inherit the discourse referents and their values from the background assignment *f*:

(38) if 〈*w* ^*′*^,*f* ^*′*^〉∈*Q*(〈*w*,*f*〉) then *f* ^*′*^⊇*f*.

An NBAS, then, is a set of pairs of the form 〈*M*,*Q*〉 with *M* a mode indicator and *Q* such a two-dimensional attitude function from possibilities to information states.

As in the systems discussed in Subsection 3.2 above, the semantic matching condition says that an ADS captures an NBAS iff every ADS compartment 〈*M*,*K*〉 is entailed by a corresponding NBAS compartment 〈*M*,*Q*〉. This time, entailment is relativized to a relevant background state, determined by the given dependency structure. Following Ninan’s static implementation, entailment relative to a background state is computed by looking at each possibility in that background individually, and checking entailment relative to the singleton state {〈*w*,*f*〉}, i.e., checking for each possibility 〈*w*,*f*〉 in the relevant background state whether the agent’s attitudinal state relative to 〈*w*,*f*〉 contains all the information in *K* interpreted relative to the accepted background anchor *f*. Using the notation $BG(Q,\mathcal {A})$ to denote the relevant background state for interpreting the two-dimensional attitude *Q* as situated within NBAS $\mathcal {A}$ (cf. Subsection 5.4 below for definition); and [[*K*]]^is,*f*^ to denote an anchored information state (cf. AAppendix for definition), gives the following matching condition.

(39) $\text {An ADS} \,\mathcal {K}\, \text {captures an NBAS} \,\mathcal {A}\, \text {iff there is a variable renaming function}$ $r\, \text {on} \,\mathcal {K}\, \text {such that for every} \,\left \langle M,K \right \rangle \in \mathcal {K}\, \text {there is a} \,\left \langle M,Q \right \rangle \in \mathcal {A}\, \text {such that for}$ $\text {all} \,\left \langle w,f \right \rangle \in BG(Q,\mathcal {A}): Q(\left \langle w,f \right \rangle ) \rhd {[{\kern -2.3pt}[ r(K)]{\kern -2.3pt}]}^{\textsf {is},f}.$

Essentially, (39) combines the Kampian semantic matching condition for ADT, as reconstructed in Section 3.2.3, and (a suitably generalized and dynamified version of) Ninan’s two-dimensional semantics, as reconstructed in Section 5.1.

### Example 1: Ninan’s Puzzle

Before we delve into the general definition of attitudinal backgrounds, let me illustrate the system so far by applying it to the *de dicto* version of Ninan’s puzzle that we used as a counterexample to Kamp’s system above.

(40)

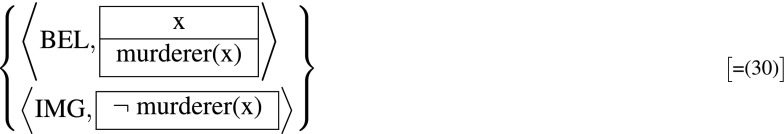

As before, we’ll call the belief DRS *K*_1_ and the imagination *K*_2_. According to (39), this ADS captures someone’s mental state given as an NBAS $\mathcal {A}$ if the NBAS contains a belief 〈BEL,*Q*_1_〉 and an imagination 〈IMG,*Q*_2_〉 such that (i) relative to any possibility in the belief’s background, *Q*_1_ entails *K*_1_ and (ii) relative to any possibility in the imagination’s background, *Q*_2_ entails *K*_2_. Formally:

(41) The ADS *K* in (40) captures NBAS *A* iff a. for all 〈*w*,*f*〉∈*B* *G*(*Q*_1_,*A*): *Q*_1_(〈*w*,*f*〉)▹[[*K*_1_]]^is,*f*^ b. for all 〈*w*,*f*〉∈*B* *G*(*Q*_2_,*A*):*Q*_2_(〈*w*,*f*〉)▹[[*K*_2_]]^is,*f*^

Intuitively, in this particular example it’s clear what the relevant background states are. The belief DRS is referentially independent in the ADS, so let’s assume its semantic counterpart *Q*_1_ in the NBAS is independent as well. This means that $BG(Q_{1}, \mathcal {A})$ is empty, i.e., the empty information state, Λ(={〈*w*,*∅*〉*w*∈*W*}), not the empty set. Filling that in in (41a) gives:

(42) for all *w*∈*W*:*Q*_1_(〈*w*,*∅*〉)▹[[*K*_1_]]^is^ [⇔(41a)]

In words: *Q*_1_, relative to any possible world, must contain at least all the information in the belief DRS *K*_1_, i.e., that there is a murderer x. Now recall that we assumed that *Q*_1_ was independent. If it is really independent, then, intuitively, it should determine the same state relative to any background possibility, i.e., the Ninan-style two-dimensional background-relativity should be vacuous. We’ll make this more precise below, but for now we’ll assume that *Q*_1_(〈*w*,*∅*〉) = *Q*_1_(〈*w* ^*′*^,*∅*〉) for any *w*,*w* ^*′*^∈*W*. We’ll use the notation $\overline {Q_{1}}$ to denote the belief state determined by *Q*_1_ relative to an arbitrary 〈*w*,*∅*〉. Thus, the matching requirement for the belief is that $\overline {Q_{1}}$ should contain the information that there is a murderer x.

The imagination DRS *K*_2_ is referentially dependent on the belief DRS, so its semantic counterpart *Q*_2_ should be dependent on the belief’s counterpart *Q*_1_. Given *Q*_1_’s independence, this means that $BG(Q_{2},\mathcal {A}) = \overline {Q_{1}}$. Filling this in in (41b):

(43) $\text {for all} \,\left \langle w,f \right \rangle \in \overline {Q_{1}}: Q_{2}(\left \langle w,f \right \rangle )\rhd {[{\kern -2.3pt}[ K_{2}]{\kern -2.3pt}]}^{\textsf {is},f}$ [ ⇔(41b)]

Matching the imagination thus requires that *Q*_2_, relative to any belief-possibility 〈*w*,*f*〉, entails the informational content of *K*_2_ relative to *f*. Now, any $\left \langle w,f \right \rangle \in \overline {Q_{1}}$ is such that x∈*D* *o* *m*(*f*) (and, moreover, *f*(*x*) is a murderer in *w*), so using such an *f* as anchor fixes the reference of the free variable x in *K*_2_: ${[{\kern -2.3pt}[ K_{2}]{\kern -2.3pt}]}^{\textsf {is},f} = \{\left \langle w^{\prime },f \right \rangle \vline f(\mathrm {x})\not \in \mathbb {I}_{w^{\prime }}(\text {murderer})\}$. Relative to any belief-possibility 〈*w*,*f*〉, wherein *f*(x) is a murderer, the content of the imagination is that *f*(x) is not a murderer. This captures precisely the nature of Ninan’s counterfactual imagination.

I conclude that Ninan’s two-dimensional solution to his problem of counterfactual *de re* attitudes carries over to the more powerful dynamic ADT framework. Incorporating Ninan’s insight into ADT crucially involves moving from ISBAS, representing attitudes as context change potentials, to NBAS, representing attitudes as information states relative to a background possibility.

In the remainder of the paper I make the determination of the relevant backgrounds for components of an NBAS more precise. I’ll thereby present an ADS semantics that is as general as Kamp’s with respect to modeling parasitic attitudes (e.g., allowing multiple simultaneous and recursive dependencies), but that avoids the problems of counterfactual attitudes that I brought out in Section 4.

### Determining Background States

The question that is not yet answered in sufficient detail and generality above is: How do we determine the relevant background state, $BG(Q,\mathcal {A})$, for an arbitrary attitude *Q* in an arbitrary NBAS $\mathcal {A}$? The idea is that the relevant attitudinal background for interpreting a given dependent attitude should be determined on the basis of the dependency structure in the NBAS.

The first hurdle is that, unlike with Kamp’s CCPs, in the current setup we can’t compute a semantic dependency relation purely from the attitudes in the NBAS, because every attitude is simply a function from possibilities to information states. We’ll add a primitive semantic dependency relation ≺^∗^ to the NBAS and demand, in the matching condition, that it mirrors the syntactic dependency ≺. Formally, this requires adding some bookkeeping to the matching condition:^12^

(44) $\text {An ADS} \,\mathcal {K} \,\text {captures an NBAS} \,\left \langle \mathcal {A}, \prec ^{*} \right \rangle \, \text {iff there is a variable renaming function}$ $r\, \text {on} \,\mathcal {K}\, \text {and a one-to-one function} \,c\, \text {from} \,\mathcal {K}\, to \,\mathcal {A}\, \text {such that for every} \,\left \langle M,K \right \rangle \in \mathcal {K}:$
  - $\label {aaa} c(\left \langle M,K \right \rangle ) = \left \langle M,c_{2}{\left \langle M,K \right \rangle } \right \rangle \in \mathcal {A}$
  - $\text {for every}\, \left \langle M^{\prime },K^{\prime } \right \rangle \in \mathcal {K}: \text {if} \,K\prec K^{\prime } \,\text {then} \,c_{2}(\left \langle M,K \right \rangle )\prec ^{*} c_{2}(\left \langle M^{\prime },K^{\prime } \right \rangle )$
  - $\text {for every} \,\left \langle w,f \right \rangle \in BG(c_{2}(\left \langle M,K \right \rangle ), \mathcal {A}): (c_{2}(\left \langle M,K \right \rangle ))(\left \langle w,f \right \rangle ) \rhd {[{\kern -2.3pt}[{r(K)}]{\kern -2.3pt}]}^{\textsf {is},f}.$

With ≺^∗^ we can determine for each attitude *Q* in $\mathcal {A}$ the set of attitudes it depends on:

(45) $Deps(Q,\left \langle \mathcal {A},\prec ^{*} \right \rangle ) = \left \{Q^{\prime }\ \vline \ \text {there is an}\, M^{\prime } \,\text {such that }\,\left \langle M^{\prime },Q^{\prime } \right \rangle \in \mathcal {A}\, \text {and} \, Q^{\prime }\prec ^{*} Q\right \}$

We call an attitude ≺^∗^-independent if its set of dependencies, as defined in (45), is empty. If *Q* is independent, the Ninan-style background-relativity built into *Q* by the general definition of attitudes as two-dimensional functions, should be vacuous. We enforce this connection between (Kamp-style) ≺^∗^-dependence and (Ninan-style) two-dimensional dependence as follows:

(46) If Q is ≺^∗^-independent in $\left \langle \mathcal {A}, \prec ^{*} \right \rangle ,$ then: *Q*(〈*w*,*f*〉) = *Q*(〈*w* ^*′*^,*f* ^*′*^〉), for all possibilities 〈*w*,*f*〉,〈*w* ^*′*^,*f* ^*′*^〉 with *D* *o* *m*(*f*) = *D* *o* *m*( *f* ^*′*^).

Now, finally we can state the general definition of *BG*. The idea is that the background of an independent attitude is the empty information state, while the background of a dependent attitude is the sum of the attitudinal states it depends on. However, these attitudinal states may in turn depend on yet other backgrounds, which introduces recursion into the definition. The full definition is in (47), an example illustrating how it works follows in Section 5.5 below.

(47) $BG(Q,\left \langle \mathcal {A},\prec ^{*} \right \rangle ) = \\ \left \{\begin {array}{l} = \Lambda ,\ \text {if} \,Q\,\, \text {is independent;}\\ = \bigcup \left \{Q_{1}(i_{1})\uplus {\ldots } \uplus Q_{n}(i_{n})\ \vline \ i_{1}\in BG(Q_{1}, \left \langle \mathcal {A}, \prec ^{*} \right \rangle ){\ldots } i_{n}\in BG(Q_{n}, \left \langle \mathcal {A}, \prec ^{*} \right \rangle )\right \}\\ {\kern 156pt} , \text {if} \,Deps(Q,\left \langle \mathcal {A}, \prec ^{*} \right \rangle )=\left \{Q_{1}{\ldots } Q_{n}\right \} \end {array}\right .$

### Example 2: Multiple and Recursive Dependencies

Let me illustrate my proposal with the abstract example from (26), repeated below:

(48) Example ADS:{〈 BEL,*K*_1_〉,〈 BEL,*K*_2_〉,〈 DES,*K*_3_〉,〈 IMG,*K*_4_〉}, with *K*_1_≺ *K*_4_ and *K*_2_≺*K*_3_≺*K*_4_.[=(26)]

To satisfy the matching condition in (44) we need an NBAS $\left \langle \mathcal {A},\prec ^{*} \right \rangle $ with matching intensional attitude components and a parallel dependency structure. As in the previous systems, a matching NBAS for this ADS could in principle have many more components, but for simplicity let’s consider an NBAS with the same cardinality and dependency structure as the ADS:

(49) Example NBAS:{〈 BEL,*Q*_1_〉,〈 BEL,*Q*_2_〉,〈 DES,*Q*_3_〉,〈 IMG,*Q*_4_〉}, with *Q*_1_≺^∗^ *Q*_4_ and *Q*_2_≺^∗^ *Q*_3_≺^∗^ *Q*_4_.

The first part of the matching condition, i.e. (44a) and (44b), is now trivially satisfied by choosing a one-to-one function *c* with *c*(〈*M*,*K*_*i*_〉)=〈*M*,*Q*_*i*_〉. We won’t worry about renaming variables and assume that the discourse referents in the ADS and the NBAS already match. The remaining, crucial requirement of the matching condition is this:

(50) $\text {For every} \,\left \langle w,f \right \rangle \in BG(Q_{i},\left \langle \mathcal {A}, \prec ^{*} \right \rangle ): Q_{i}(\left \langle w,f \right \rangle ) \rhd {[{\kern -2.3pt}[ K_{i}]{\kern -2.3pt}]}^{\textsf {is},f} (i=1\ldots 4).$

Roughly, each attitude *Q*_*i*_ in $\mathcal {A}$, relative to any relevant background possibility, must entail the information state expressed by the corresponding, anchored attitude DRS.

To figure out what that means we need to compute the relevant backgrounds for each attitude. For the independent attitudes, the two beliefs, the background is the empty information state. For readability I suppress the constant $\langle {\mathcal {A},\prec }\rangle $ arguments from here on.

(51) *B* *G*(*Q*_1_) = *B* *G*(*Q*_2_)=Λ

The desire *Q*_3_ depends on the second belief *Q*_2_, so its background is the information state given by *Q*_2_. Formally, however, *Q*_2_ is a function that needs a possibility argument to yield an information state. Since *Q*_2_ is independent, its two-dimensional background-relativity is vacuous (by (46)). Using the notational shorthand introduced in Subsection 5.2: $\overline {Q}\ (= Q(\left \langle w,\emptyset \right \rangle )$ for arbitrary *w*) represents the agent’s independent belief state. Filling in the definition of backgrounds in (47) gives:

(52) $BG(Q_{3}) = \bigcup \left \{Q_{2}(i)\ \vline \ i\in \Lambda \right \}= \overline {Q_{2}}$

In words, the background state for *Q*_3_ is the information state determined by the independent belief *Q*_2_.

The imagination *Q*_4_ depends on two attitudes, hence its background requires merging the information states of those attitudes, relative to their backgrounds:

(53) $BG(Q_{4}) = \bigcup \left \{Q_{3}(i_{1})\uplus Q_{1}(i_{2})\ \vline \ i_{1}\in BG(Q_{3}), i_{2}\in \Lambda \right \}$ ${\kern 184pt} = \bigcup \left \{Q_{3}(i_{1})\uplus \overline {Q_{1}}\ \vline \ i_{1}\in \overline {Q_{2}}\right \}$

In words, *B* *G*(*Q*_4_) is the set of possibilities *i* in the merge of the independent belief $\overline {Q_{1}}$ and the desire *Q*_3_ relative to *i* ^*′*^, for some *i* ^*′*^ in the independent belief state $\overline {Q_{2}}$.

Note the use of information state merging, ⊎, in (53), which I borrow straight from Kamp’s semantics: in case of multiple simultaneous dependencies, the background attitudes are merged together into a single, blended background information state. As a result, in our example we only get a meaningful imagination background if the belief state $\overline {Q_{1}}$ is consistent with (some of) the relative desire states *Q*_3_(*i* ^*′*^).^13^ Recall that, in fact, Kamp’s system further required consistency between dependent attitudes and their backgrounds. It is this requirement that I showed to be problematic in Section 4, and which we set out to solve here.

Having now computed the four background states we can return to the matching condition, (50) for our example. Filling in the blanks gives:

(54) The ADS in (48) captures the NBAS in (49) iff
  - For every 〈*w*,*f*〉∈Λ:*Q*_1_(〈*w*,*f*〉)▹[[*K*_1_]]^is,*f*^
  - For every 〈*w*,*f*〉∈Λ:*Q*_2_(〈*w*,*f*〉)▹[[*K*_2_]]^is,*f*^
  - $\text {For every}\, \left \langle w,f \right \rangle \in \overline {Q_{2}} : Q_{3}(\left \langle w,f \right \rangle ) \rhd {[{\kern -2.3pt}[ K_{3}]{\kern -2.3pt}]}^{\textsf {is},f}$
  - $\text {For every}\, \left \langle w,f \right \rangle \in \bigcup \{Q_{3}(i)\uplus \overline {Q_{1}}\ \vline \ i\in \overline {Q_{2}}\}: Q_{4}(\left \langle w,f \right \rangle ) \rhd {[{\kern -2.3pt}[{K_{4}}]{\kern -2.3pt}]}^{\textsf {is},f}$

In words, (54a) and (54b) simply say that the two independent belief states in the NBAS entail the corresponding belief DRSs. We can bring this out by rewriting (54a,b) using our overline notation:

(55)
  - $\overline {Q_{1}} \rhd {[{\kern -2.3pt}[ K_{1}]{\kern -2.3pt}]}^{\textsf {is}} \qquad \qquad \quad \,\,\qquad \qquad \qquad \qquad \qquad \qquad \qquad \qquad \left [\Leftrightarrow (54\text {a})\right ]$
  - $\overline {Q_{2}} \rhd {[{\kern -2.3pt}[{K_{2}}]{\kern -2.3pt}]}^{\textsf {is}} \qquad \qquad \quad \,\,\qquad \qquad \qquad \qquad \qquad \qquad \qquad \qquad \left [\Leftrightarrow (54\text {b})\right ]$

As for the desire, (55c) says that the two-dimensional desire *Q*_3_ entails the desire DRS *K*_3_ relative to every possibility in the belief *Q*_2_. This makes sense given that in $\mathcal {K}$ the desire *K*_3_ referentially depends on the belief *K*_2_.

Finally, because of the recursive, multiple dependence, (55d) is not as easy to read. It’s contribution may be brought out more clearly by rewriting it equivalently as follows:

(56) $\text {For every} \,\left \langle w,f \right \rangle \in \overline {Q_{1}}\, \text {and} \,\left \langle w^{\prime },f^{\prime } \right \rangle \in Q_{3}(\left \langle w,f \right \rangle )\uplus \overline {Q_{1}}:\,Q_{4}(\left \langle w^{\prime },f^{\prime } \right \rangle ) \rhd {[{\kern -2.3pt}[{K_{4}}]{\kern -2.3pt}]}^{\textsf {is},f^{\prime }}$ [⇔(54d)]

## Conclusion

The first, negative contribution of this paper consists in demonstrating that Kamp’s semantic analysis of mental states falls prey to Ninan’s puzzle and variations thereof. After carefully reconstructing Kamp’s Attitude Description Theory (ADT), I show that the system fails to account for referential dependencies between inconsistent attitudes. This is a serious limitation, because so-called counterfactual attitudes, like imagining, pretending and wishing, are typically inconsistent with the beliefs on which they depend.

In my reconstruction of Kamp’s system we see that the problem results from the use of context change potentials as the primitive model-theoretic representations of individual attitude contents. Based on this diagnosis I propose a solution where, following an idea from Ninan, attitudes are given relative to an assumed background.

The current paper contributes to our understanding of human cognition in general by developing a model of mental states as complex networks of interconnected attitudes in a precise logical framework. Moreover, this ADT model has important applications in linguistics. In [20], for instance, I use Kampian mental state representations to analyze Karttunen’s [17] puzzle about presupposition resolution across attitude ascription complements, and Pross [23] uses them to analyze Fodor’s [5] ‘third readings’ of attitude reports. But the impact of ADT extends beyond the semantics of attitudes and reports. The ADT framework is a foundational component of Kamp’s program of developing a communication-theoretic analysis of natural language meaning in general. In his recent work Kamp has been arguing for a revival of the classic sender–receiver model of communication in formal semantics. On this view, semantics is not, or not only, about deriving participant-neutral meanings, such as objective truth conditions or potentials to update the common ground, but rather involves formulating algorithms capturing the production and comprehension of discourse by cognitive agents. Concretely, the speaker has a certain mental state, modeled as an ADS, and wants to map that to an utterance. The hearer, by contrast, takes an utterance as input and uses that to update her mental state, again modeled as an ADS. A representational semantic analysis of mental states, with a precise model-theoretic interpretation, such as that provided by the current modification of Kamp’s ADT, is thus a first step in this new direction of natural language semantics in general.

## Appendix

### Discourse Representation Theory (DRT)

(57) primitive symbols of DRS language
  - a set of predicates *P* *r* *e* *d*={P,Q,walk,john,beat,=,…}
  - a set of discourse referents *D* *R* *e* *f*={x,y,x_1_,…}

(58) syntax of DRS language
  - if *x*_1_…*x*_*n*_ are discourse referents and *P* *an* *n* -place predicate, then *P*(*x*_1_…*x*_*n*_) is a DRS condition
  - if *x*_1_…*x*_*n*_ are discourse referents and *ψ*_1_…*ψ*_*m*_ are DRS conditions, then 〈{*x*_1_…*x*_*n*_},{*ψ*_1_…*ψ*_*m*_}〉 is a DRS (notation: *U*(*K*)−{*x*_1_…*x*_*n*_} and *C* *o* *n*(*K*)={*ψ*_1_…*ψ*_*m*_})
  - if *K*,*K* ^*′*^ are DRSs, then ¬*K* and *K*⇒*K* ^*′*^ are DRS conditions

(59) free variables of a DRS/condition:
  - $FV(K) = \bigcup \left \{FV(\psi )\ \vline \ \psi \in Con(K)\right \}\backslash U(K)$
  - *F* *V*(*P*(*x*_1_…*x*_*n*_))={*x*_1_…*x*_*n*_}
  - *F* *V*(¬*K*) = *F* *V*(*K*)
  - *F* *V*(*K*⇒*K* ^*′*^) = *F* *V*(*K*)∪(*F* *V*(*K* ^*′*^)∖*U*(*K*))
  - *K* is proper if *F* *V*(*K*) = *∅*, improper otherwise.

(60) notation: *f*⊆_*X*_ *g*: = *f*⊆*g*∧*D* *o* *m*(*g*) = *D* *o* *m*(*f*)∪*X*

(61) intensional model: $M = \left \langle D,W,\mathbb {I} \right \rangle ,\, \text {where}\, \mathbb {I} : W\times Pred \rightarrow \bigcup \nolimits _{n\in \mathbb {N}}D^{n}$

(62) set of embeddings *F*: *f*∈*F* iff *f* is a partial function from *D* *R* *e* *f* to *D*

(63) embeddings verifying a DRS/condition, relative to a model *M* (note: I suppress the *M* in notation throughout):
  - *g*⊧_*w*_ *K* if for all *ψ*∈*C* *o* *n*(*K*):*g*⊧_*w*_ *ψ*
  - $g\models _{w} P(x_{1},{\ldots } x_{n})\, \text {if} \,\left \langle g(x_{1}){\ldots } g(x_{n}) \right \rangle \in \mathbb {I}_{w}(P)$
  - *g*⊧_*w*_¬*K* if there is no *h*⊇_*U*(*K*)_ *g* with *h*⊧_*w*_ *K*.

(64) static interpretation of proper DRS *K*:
  - [[*K*]]^*f*^={*w*∈*W* ∃*g*⊇_*U*(*K*)_ *f*[*f*⊧_*w*_ *K*]}
  - [[*K*]]=[[*K*]]^*∅*^

(65) $\text {set of information states} \,\mathcal {I}:$ $s\in \mathcal {I} \,\mathit {iff}$
  - *s*⊂*W*×*F*
  - there is a *Y*⊂*D* *R* *e* *f* such that: ∀〈*w*,*f*〉∈*s*[*D* *o* *m*(*f*) = *Y*] (notation: Y = *Base(s)*, the base of *s*)

(66) $\text {possibilities (notation): if}\, i\in s\in \mathcal {I},\, \text {then}\, i=\left \langle w_{i},f_{i} \right \rangle $

(67) empty information state: Λ={〈*w*,*∅*〉 *w*∈*W*}

(68) information state interpretation of proper DRS *K*:
  - [[*K*]]^is,*f*^={〈*w*,*g*〉 *g*⊇_*U*(*K*)_ *f* and *g*⊧_*w*_ *K*}
  - [[*K*]]^is^=[[*K*]]^is^^*∅*^

(69) set of regular context change potentials $\,\mathcal {J}:$ $j\in \mathcal {J}\, \text {iff}$
  - $j\, \text {is a partial function from} \,\mathcal {I}\, to \,\mathcal {I}$
  - there is a *Y*⊂*D* *R* *e* *f* such that: *s*∈*D* *o* *m*(*j*) iff *Y*⊆*B* *a* *s* *e*(*s*) (notation: *Y* = *P* *r* *e* *s*(*j*) ,the referential presuppositions of *j* )
  - there is a *Z*⊂*D* *R* *e* *f* such that: if *s*∈*D* *o* *m*(*j*) ,then *B* *a* *s* *e*(*j*(*s*)) = *B* *a* *s* *e*(*s*)∪ *Z* (notation: *Z* = *B* *a* *s* *e*(*j*),the base of *j*)
  - $j(s)=\bigcup \nolimits _{i\in s}j(\left \{i\right \})$

(70) dynamic/CCP interpretation of DRS *K*: ${[{\kern -2.3pt}[ K]{\kern -2.3pt}]}^{\textsf {ccp}}\in \mathcal {J}\, \text {and} \,{[{\kern -2.3pt}[{K}]{\kern -2.3pt}]}^{\textsf {ccp}}(s)= \{\left \langle w,f \right \rangle \ \vline \ \exists g [\left \langle w,g \right \rangle \in s\, \text {and} \,f\supseteq _{U(K)} g\, \,\text {and} \,f\models _{w} K]\},$ if *F* *V*(*K*)⊆*B* *a* *s* *e*(*s*); undefined otherwise.

(71) *s* contains a least as much information as *s* ^*′*^: *s*▹*s* ^*′*^ iff for all 〈*w*,*f*〉∈*s* there is a *g*⊆*f* such that 〈*w*,*g*〉∈*s* ^*′*^.

(72) merge of information states: *s*⊎*s* ^*′*^={〈*w*,*f*∪*f* ^*′*^〉 〈*w*,*f*〉∈*s* and〈*w*,*f* ^*′*^〉∈*s* ^*′*^ and *f*∪*f* ^*′*^ is a function}

### Attitude Description Theory (ADT)

(73) *K* ^*′*^ referentially depends on *K*: *K*≺*K* ^*′*^ iff *F* *V*(*K* ^*′*^)∩*U*(*K*)≠*∅*

(74) $\text {dependencies of an attitude DRS}\, K\, \text {in an ADS}\, \mathcal {K} :$ $Deps(K,\mathcal {K}) = \left \{K^{\prime }\ \vline \ \exists l [\left \langle l,K^{\prime } \right \rangle \in \mathcal {K} \wedge K^{\prime }\prec K\right ]\}$

(75) $\text {information state expressed by an attitude DRS} \,K\, \text {in an ADS}\, \mathcal {K}:$ ${[{\kern -2.3pt}[{K}]{\kern -2.3pt}]^{\textsf {is}}_{\mathcal {K}}}= \left \{\begin {array}{ll} {[{\kern -2.3pt}[ K]{\kern -2.3pt}]}^{\textsf {is}}& \text {if} \,K\, \text {is proper} \\\\ {[{\kern -2.3pt}[ K]{\kern -2.3pt}]}^{\textsf {ccp}}({[{\kern -2.3pt}[ K_{1}]{\kern -2.3pt}]}^{\textsf {is}}_{\mathcal {K}}\uplus \ldots \uplus {[{\kern -2.3pt}[{K_{n}}]{\kern -2.3pt}]}^{\textsf {is}}_{\mathcal {K}})& \text {if} \,K\, \text {improper and} \\ &Deps(K,\mathcal {K}) = \left \{K_{1}{\ldots } K_{n}\right \} \end {array}\right .$

(76) referential dependence for CCPs: *j*≺^∗^ *j* ^*′*^ iff *P* *r* *e* *s*(*j* ^*′*^)∩*B* *a* *s* *e*(*j*)≠*∅*

(77) $\text {dependencies of an attitude CCP}\, j\, \text {in an ISBAS}\, \mathcal {A} :$ $Deps(j,\mathcal {A}) = \left \{j^{\prime }\in \mathcal {A}\ \vline \ j^{\prime }\prec ^{*} j\right \}$

(78) $\text {information state associated with} j\, \text {in ISBAS}\, \mathcal {A}$ $IS(j,\mathcal {A}) = \left \{\begin {array}{ll} j(\Lambda ) &\text {if} \,j\, \text {is total}\\\\ j(IS(j_{1},\mathcal {A})\uplus \ldots \uplus IS(j_{n},\mathcal {A}))& \text {if} \,j\, \text {is non-total and} \\& Deps(j,\mathcal {A})=\left \{j_{1}{\ldots } j_{n}\right \} \end {array}\right .$

## Footnotes

1 I’m simply quantifying into the intensional environment here. See the discussion of Ninan’s puzzle below for a more refined analysis of *de re* (and its problems).

2 For some recent discussion of this puzzle see Elbourne [4], Schoubye [25].

3 For some recent discussion of Karttunen’s puzzle and Heim’s semantics, see Villalta [27], Maier [20].

4 I’ll be using the indexical i to represent the first-person or *de se* center of an attitude. Formally, that means that the proposition it occurs in should be viewed as a “centered proposition”. In this case that means that the attitude operator BEL quantifies over centered worlds. The formula in (3) means that all centered belief alternatives 〈*w*,*s*〉 are such that the center *s* is uniquely R-acquainted with some x, and that x has property P.

5 For some recent discussion of Ninan’s puzzle, see Yanovich [22], Ninan [28].

6 The construction of a (not quite compositional) semantics of attitude reports, along the lines suggested here, is the subject of another paper [20]. In that paper I do the opposite, viz. focus on the linguistics and simplify the underlying theory of mental states.

7 Of course we can make ad hoc stipulations to deal with these specific cases. For instance, we could treat (9a) as just a conjunction. This is ad hoc because surely we wouldn’t want to say that any discourse, say a novel, or a long conversation between multiple people, consists of just one sentence, which cannot be interpreted until it’s completed. Similarly, for (9b) we could say that *a farmer* is ambiguous between an existential and a universal reading. Perhaps the least ad hoc way of dealing with the examples in (9) in a traditional, static framework is the E-type approach, where pronouns are not variables but abbreviated definite descriptions (cf. Heim [9]).

8 My presentation of ADT deviates from Kamp’s. In particular, I added the static PBAS system below in order to motivate the dynamic approach.

9 Or rather, to take *de se* attitudes into account, centered propositions, but I try to abstract away from that issue here.

10 For more on the philosophical motivations and linguistic applications of information states and dynamic semantics, cf. Dekker [2].

11 I’m still abstracting away from orthogonal issues surrounding *de se* attitudes. Ninan’s implementation uses centered worlds/propositions.

12
*c*_2_ is the projection of the output of *c* on the second coordinate.

13 As far as I can tell this prediction/constraint, which I share with Kamp’s original proposal, is unproblematic. If counterexamples should arise, a possible solution to explore in future work could involve a more sophisticated merge operation using ideas from belief revision to deal with inconsistencies (Gärdenfors [6]).
